# Compositional Analysis and Aroma Evaluation of Feijoa Essential Oils from New Zealand Grown Cultivars

**DOI:** 10.3390/molecules24112053

**Published:** 2019-05-29

**Authors:** Yaoyao Peng, Karen Suzanne Bishop, Siew Young Quek

**Affiliations:** 1 Food Science, School of Chemical Sciences, The University of Auckland, Auckland 1010, New Zealand; ypen083@aucklanduni.ac.nz; 2 Discipline of Nutrition and Dietetics, School of Medical Science, Faculty of Medicine and Health Science, The University of Auckland, Auckland 1023, New Zealand; k.bishop@auckland.ac.nz; 3 Riddet Institute, New Zealand Centre of Research Excellence for Food Research, Palmerston North 4474, New Zealand

**Keywords:** feijoa essential oil, hydro-distillation, GC-MS, HS-SPME-GC-O-MS, aroma active compounds, aroma profile

## Abstract

Feijoa is an aromatic fruit and the essential oil from feijoa peel could be a valuable by-product in the juicing industry. An initial comparison of the essential oil extraction methods, steam-distillation and hydro-distillation, was conducted. The volatile compounds in the essential oils from four feijoa cultivars were identified and semi-quantified by GC-MS and the aroma active compounds in each essential oil were characterized using SPME-GC-O-MS. Hydro-distillation, with a material to water ratio of 1:4 and an extraction time of 90 min, was the optimized extraction method for feijoa essential oil. The Wiki Tu cultivar produced the highest essential oil yield among the four selected cultivars. A total of 160 compounds were detected, among which 90 compounds were reported for the first time in feijoa essential oils. Terpenes and esters were dominant compounds in feijoa essential oil composition and were also major contributors to feijoa essential oil aroma. Key aroma active compounds in feijoa essential oils were *α*-terpineol, ethyl benzoate, (*Z*)-3-hexenyl hexanoate, linalool, (*E*)-geraniol, 2-undecanone, 3-octanone, *α*-cubebene, and germacrene D. This is the first report on the optimization of the extraction method and the establishment of the aroma profile of feijoa essential oils, with a comparison of four New Zealand grown cultivars.

## 1. Introduction

Essential oils are aromatic volatile liquids which can be extracted from various plant materials, including flowers, leaves, herbs, and fruits. The history of essential oil extraction and application can be traced back to the Middle Ages [1]. Due to the extremely low extraction yield, essential oils are often deemed as ‘liquid gold’.

Plant derived essential oils have been widely applied in the food, perfume, pharmaceutical, and aromatherapy industries [1,2,3,4]. For instance, essential oils from oregano, cinnamon, thyme, and rosemary are employed in food packaging to extend shelf life [5,6,7,8], lavender essential oils are famous for their relaxing effects [9,10], and citrus essential oils are reported to have antimicrobial, antioxidant, anti-inflammatory, and anti-cancer properties [11,12,13,14]. Particularly in the flavor and fragrance industries, compared with artificial compounds, natural essential oils could be a safer choice for food flavoring and hygiene products.

Feijoa (*Acca sellowiana* (O. Berg) Burret) is a subtropical fruit with intense aroma. It was originally discovered in South America [15] and is now widely grown in New Zealand. The fruit has a green skin and a jelly pulp with a sweet-sour taste. The unique aroma/flavor of feijoa fruit is one of its most characteristic features and is believed to contribute greatly to its increasing consumption. However, very limited research has focused on volatile compounds in feijoa fruit and feijoa essential oil and the aromatic properties of feijoa have not been thoroughly reported. Hardy and Michael [16] are among the earliest researchers of feijoa volatiles and they found that methyl benzoate and ethyl benzoate were dominant aroma active volatile compounds, accounting for over 90% of the total feijoa volatile oil. Despite this early publication, to date, fewer than five published works have mentioned the extraction and component analysis of feijoa essential oil and the aroma active compounds largely remain unknown.

Cultivar variation exists in many well-known fruits, such as apple [17], grape [18], and strawberry [19]. Cultivar may affect fruit size, color, texture, aroma, flavor, and nutritional and bioactive compound composition. Feijoa cultivars can be roughly divided according to their ripening season. Previous studies have reported the diversity of the compound composition as well as the aroma experience in the essential oils from different plant cultivars [20,21]. In addition, the components of essential oils could also serve as biomarkers for the identification of different cultivars [22]. Therefore, a systematic comparison of the compound composition and aroma profile of essential oils from different feijoa cultivars could be vital in feijoa cultivar selection and differentiation.

In this study, four feijoa cultivars, namely Unique (early season cultivar), Apollo (early to mid-season cultivar), Wiki Tu (mid to late season cultivar), and Opal Star (late season cultivar), were carefully selected following suggestions from local growers. This is the first report that optimized the extraction method, investigated the aroma active compounds, and established the aroma profile of feijoa essential oils, with a comparison of four New Zealand grown cultivars. Moreover, the peel of feijoa fruits is normally a waste material from the juicing industry and when consumed as a fresh fruit. Obtaining highly valuable essential oils from waste materials could therefore provide additional value to the feijoa industry.

## 2. Results and Discussion

### 2.1. Comparison of Steam-Distillation (SD) and Hydro-Distillation (HD)

**Figure 1:**
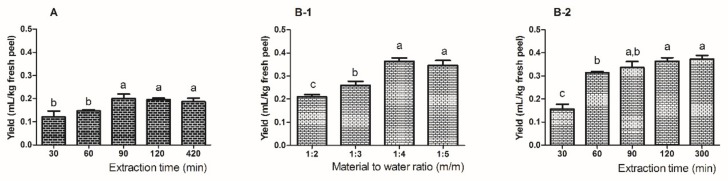
Optimization of the extraction of feijoa essential oils by steam-distillation (**A**) and hydro-distillation (**B-1**,**B-2**). (**B-1**,**B-2**) show the optimization based on the material to water ratio and extraction time, respectively. Letters a, b, c are indicators for statistical significance (*p* < 0.05), identical letters indicate no statistically significant difference.

**Figure 2:**
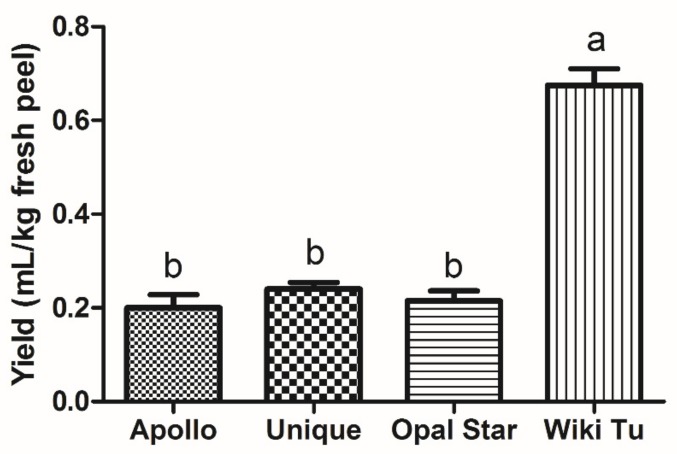
Extraction yield of feijoa essential oil from four cultivars. Letters a, b, c are indicators for statistical significance (*p* < 0.05), identical letters indicate no statistically significant difference.

The extraction of feijoa essential oil was successfully conducted by both SD and HD. As shown in Figure 1A, the essential oil yielded by SD gathered rapidly during the first 90 min of extraction and gradually become stable thereafter. With regards to HD, two parameters, material to water ratio and extraction time, were evaluated to optimize the extraction conditions. The amount of essential oil reached a peak at the material to water ratio of 1:4, although it is not significantly higher than the amount at the ratio of 1:5 (Figure 1B-1). Similar to SD, a time-dependent generation was also observed in the process of HD, in which the essential oil yield tended to reach a plateau after 90 min of extraction (Figure 1B-2). Notably, the extractable quantity of essential oil by HD was significantly higher than that by SD, indicating that HD could be more suitable than SD for the commercial extraction of feijoa essential oil. Thus, HD with a material to water ratio of 1:4 and an extraction time of 90 min was selected as the extraction method with the highest yield for feijoa essential oil.

Both SD and HD are well developed and widely applied traditional methods for the extraction of essential oils from natural plants. The history of the extraction and pharmaceutical application of essential oils can be dated back to the 13th century [1]. Previous studies have compared the extraction efficiency of SD and HD, and results have varied depending on the plant material. Boutekedjiret et al. [23] found SD was more suitable than HD for the extraction of rosemary essential oil. Kasuan et al. [24] reached a similar conclusion that SD was more efficient than HD in the extraction of essential oil from both Kaffir lime peel and leaves, while Sefidkon et al. [25] discovered a higher essential oil yield using HD than SD from *Satureja hortensis*. Similar findings from Babu and Kaul [26] were also in agreement with our present study. It has been suggested that, due to the different communication mode occurring in SD and HD, incomplete distillation may occur when materials clump together [26]. In the case of feijoa peel, significant agglutination and clumps formed during SD, which may have led to inefficient extraction.

Nevertheless, there are very few studies carried out on the extraction and analysis of feijoa essential oil. The earliest report on feijoa essential oil was by Hardy and Michael [16], who extracted feijoa volatile oil from the whole feijoa fruit by an initial HD using a rotary evaporator, followed by the re-distillation of the collected essence with trichlorofluoromethane. Following this, Shaw et al. [27] extracted essential oil from feijoa peel by low temperature vacuum SD and subsequent liquid-liquid extraction using hexane and diethyl ether. Both of the reports included organic solvents to aid in the separation of the oil phase from the aqueous phase and, unfortunately, neither of these publications mentioned the extraction yield. In a more recent study on feijoa peel essential oil, the extraction method of HD was employed and yielded 0.1% oil [28], which is consistent with our results. Despite the aforementioned studies on feijoa essential oil, our research is the first to optimize and compare the extraction efficiency of SD and HD under solvent free conditions. In addition, as evidenced from our results, feijoa peel, as a by-product from the juicing industry, has great potential for utilization in the essential oil industries.

### 2.2. Comparison of Essential Oil Yield from Four Feijoa Cultivars

The optimized HD was applied to the extraction of essential oils from four feijoa cultivars (Apollo, Unique, Opal Star, and Wiki Tu). As shown in Figure 2, the Wiki Tu cultivar produced the highest volume of essential oil, relative to the other three cultivars, reaching 0.68 mL/kg fresh peel, while no significant difference was observed among the Apollo, Unique, and Opal Star cultivars. The essential oil yield of the Wiki Tu cultivar was about three-times the amount of the other selected cultivars, which suggested that cultivar difference could significantly affect the yield of feijoa essential oils. As mentioned before, Shaw, Allen and Yates [27], Fernandez, Loiseau, Poulain, Lizzani-Cuvelier and Monnier [28] and Hardy and Michael [16] conducted essential oil extraction, while none of them compared the essential oils from different feijoa cultivars. Therefore, our research is the first to confirm cultivar difference affecting essential oil production. It may also be of interest to assess additional feijoa cultivars to select the most essential oil-rich cultivar for commercial development.

### 2.3. Compositional Analysis of Feijoa Essential Oil from Four Cultivars

The essential oils extracted from the four feijoa cultivars were subjected to compositional analysis by GC-MS. The identified compounds, with their identities and relative concentrations in the four essential oils, are summarized in Table 1. A total of 158 compounds were detected in the essential oils, by GC-MS. Among them, 89 compounds were reported in feijoa essential oil for the first time. More specifically, 114 compounds were detected in the essential oils from all four cultivars and a total of 137, 129, 148, and 141 compounds were found in the essential oils extracted from the Apollo, Unique, Opal Star, and Wiki Tu cultivar, respectively.

**Figure 3:**
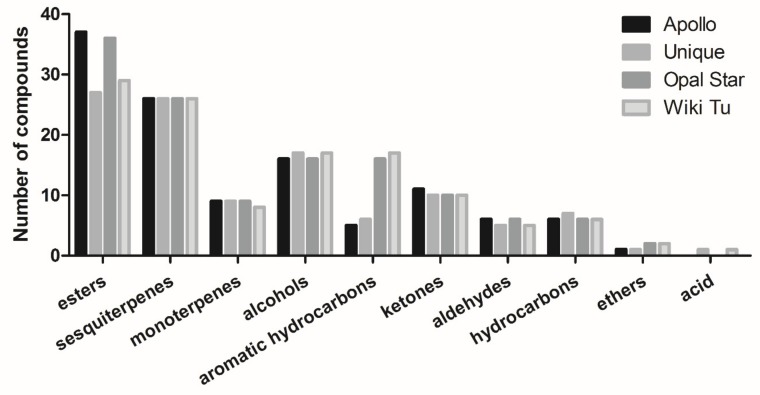
Compound distribution in essential oils from four feijoa cultivars.

As shown in Figure 3, the identified compounds (except the unknown compounds and flavone) can be classified into 10 groups. They are esters (40 compounds), sesquiterpenes (26), alcohols (19), aromatic hydrocarbons (17), ketones (11), monoterpenes (9), hydrocarbons (7), aldehydes (6), ethers (2), and an acid (1). Apollo and Opal Star essential oils comprised of more ester compounds, 37 and 36 compounds, respectively, than the Unique (27 compounds) and Wiki Tu (29 compounds) essential oils. All four essential oils contained all of the detected sesquiterpenes, but the Wiki Tu essential oil had one less monoterpene than the essential oils from the other three cultivars. The detected aromatic hydrocarbons varied significantly, reporting 5, 6, 16, and 17 compounds in the essential oils from the Apollo, Unique, Opal Star, and Wiki Tu cultivars, respectively. In addition, slight differences were seen in the composition of alcohol, ketone, aldehyde, hydrocarbon, ether, and acid compounds in the four essential oils.

The concentration of each compound differed amongst the essential oils from the four selected cultivars. For example, the concentration of 3-octanone in the Opal Star essential oil was over 30 times higher than that found in the Unique essential oil, and similarly with linalool, ethyl benzoate, (*Z*)-3-hexenyl butanoate, and (*Z*)-3-hexenyl hexanoate. The observed compositional variation among the four essential oils indicated the differences in the peel samples and could be useful to distinguish feijoa cultivars. Similar results were obtained by Xiao et al. [29], studying orange essential oil.

**Figure 4:**
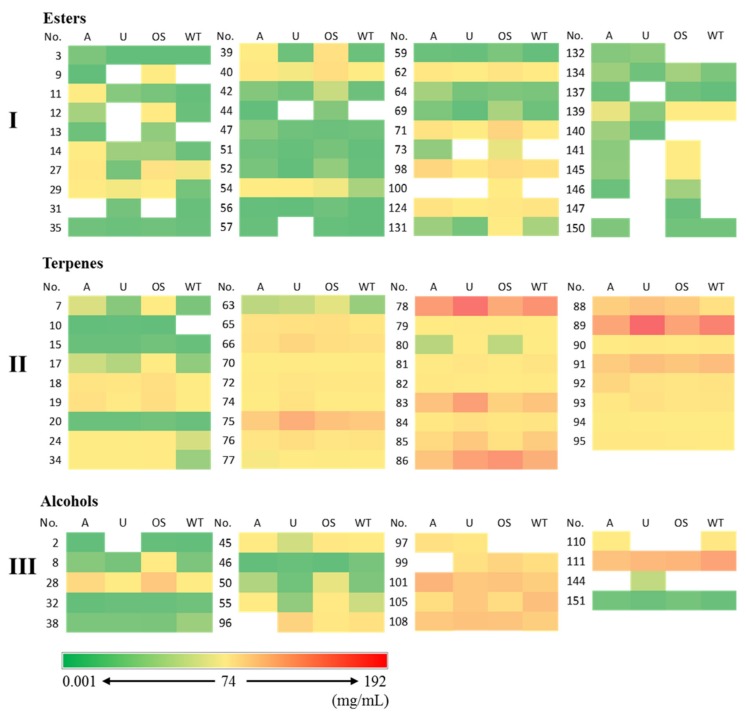
Heat map of dominant compounds in feijoa essential oils from four cultivars. (**I**)—for ester compounds; (**II**)—for terpene compounds; (**III**)—for alcohol compounds. Feijoa cultivars, A—Apollo, U—Unique, OS—Opal Star, WT—Wiki Tu. Compound numbers were consistent with those listed in Table 1.

Esters (40), terpenes (35), and alcohols (19) are the top three dominant chemical groups in feijoa essential oil composition. The most abundant compounds (Table 1 and Figure 4) in feijoa essential oil were *β*-caryophyllene (No. 78, 95.64–176.74 mg/mL), humulene (No. 83, 41.12–112.77 mg/mL), germacrene D (No. 86, 58.98–128.46 mg/mL), ledene (No. 89, 104.09–191.99 mg/mL), *α*-cadinol (No. 111, 62.22–105.78 mg/mL), *β*-elemene (No. 75, 49.19–89.72 mg/mL), and *δ*-cadinene (No. 91, 48.12–69.23 mg/mL). This is generally consistent with a previous report [28]. It is interesting to note that all these compounds, except *α*-cadinol (alcohol), were terpenes, indicating that terpenes, which are frequently found to be major components in plant essential oils in general [29,30], were dominant compounds in feijoa essential oils.

Esters were also major components (40 compounds in total) in feijoa essential oils, although the average concentration of the ester compounds was much lower than that of the terpenes (Figure 4). Important esters, of which the concentrations were above 1 mg/mL (Table 1 and Figure 4), were (*Z*)-3-hexenyl butanoate (No. 40, 1.71–21.38 mg/mL), methyl geranate (No. 62, 3.08–12.76 mg/mL), (*Z*)-3-hexenyl hexanoate (No. 71, 2.28–35.98 mg/mL), (*Z*)-3-hexenyl benzoate (No. 98, 8.11–33.53 mg/mL), and benzyl benzoate (No. 124, 2.71–14.28 mg/mL). These compounds were also reported in previous studies [27,28] on feijoa essential oil. However, it is noted that methyl benzoate and ethyl benzoate, both of which were predominant volatiles in feijoa fruits [16,31,32], were not found to be major components in feijoa essential oils. The concentration of methyl benzoate (No. 27) in the four essential oils varied significantly, with the lowest concentration detected in the Opal Star essential oil being 0.24 mg/mL, with a range of 1.70–15.46 mg/mL in the other three cultivars. Ethyl benzoate (No. 39) was only a minor component identified in the essential oils from the Unique and Wiki Tu cultivars (0.12 and 0.10 mg/mL, respectively). In addition, another important previously reported volatile ester in feijoa fruit, namely ethyl butanoate [33], was not detected in any of the four feijoa essential oils.

The third dominant group in feijoa essential oil composition was alcohol (Figure 3). Besides the aforementioned alcohol compound *α*-cadinol (No. 111), linalool, espatulenol, rosifoliol, and (−)-spathulenol (No. 28, 101, 105, and 108, respectively) were also observed at high concentrations (Figure 4), which is generally consistent with the former research [28]. However, alcohols were not determined as major volatile compounds in feijoa fruits [16,32,33], which suggested that these compounds may not be abundant in feijoa fruits, but were concentrated during the essential oil extraction.

More importantly, a number of dominant compounds in feijoa essential oil, including *β*-caryophyllene, humulene, *β*-elemene, *α*-cadinol, and linalool were discovered to harbor antioxidant, anti-inflammatory, antimicrobial, and anticancer activities [34,35,36,37,38], which indicates that feijoa essential oil could be bioactive and has the potential to be utilized in the pharmaceutical industry. Further investigations into the bioactivities of feijoa essential oil are, therefore, of great importance.

### 2.4. Aroma Active Compounds in Feijoa Essential Oil

Further to the compositional analysis of the essential oils from the four feijoa cultivars, the aroma active compounds of the essential oils were identified using the HS-SPME-GC-O-MS and they were characterized with aroma description and intensity. Due to the solvent free and non-destructive nature of the samples [39], the technique of HS-SPME is widely employed in the detection of aromatic volatile compounds in various matrixes [40,41,42].

As a result, a total of 97 compounds were detected by SPME-GC-MS (Table 1, compounds marked with *). A few compounds that were discovered by GC-MS liquid injection were not detected. These compounds were either at extremely low concentrations or had a high boiling point. In particular, the compounds with a retention index (RI) larger than 1847 (after No. 131 in Table 1) were not extracted by SPME. Similar findings from Pripdeevech, Khummueng and Park [42] working on agarwood essential oil were observed. To the contrary, two compounds, 3-octanyl acetate and *β*-selinene, were only detected by SPME-GC-MS, which could be due to the fact that SPME concentrates volatile compounds, enabling the detection of the compounds at trace amounts [39]. Therefore, a total of 160 compounds were detected in feijoa essential oils by both GC-MS and SPME-GC-MS and 90 of them were reported for the first time.

As shown in Table 2, 24 aroma active compounds consisting of eight esters, eight terpenes, three alcohols, two ketones, and three unknown compounds were identified by olfactory tests. The four chemical groups were also major compositional classes in feijoa essential oil (Figure 3). The most important aroma active compound, which was consistently detected with ‘strong’ intensity (aroma intensity > 4) in the four essential oils, was the ‘fresh and minty’ *α*-terpineol. Other major aroma contributors, with an average intensity above 3 in all essential oils, were the ‘fresh and fruity’ ethyl benzoate, the ‘honey-like’ (*Z*)-3-hexenyl hexanoate, the ‘floral’ linalool, the ‘fruity and peach-like’ (*E*)-geraniol, the ‘bitter and herbal’ 2-undecanone, the ‘mushroom-like’ 3-octanone, the ‘sweet and herbal’ *α*-cubebene, the ‘sweet, herbal, and floral’ germacrene D, and two of the unknown compounds identified with the ‘bitter and pungent’ and the ‘fruity and honey-like’ notes (RI of 1153 and 1367, respectively, in Table 1).

However, the essential oils from the four feijoa cultivars were found to consist of various aroma active compounds. The ‘banana-like’ (*Z*)-3-hexenyl acetate was only detected in the Apollo and Wiki Tu essential oils, the ‘fresh and minty’ (4*E*,6Z)-allo-ocimene were not perceived in the Apollo and Unique essential oils, and the ‘sweet and herbal’ (*E*)-*β*-ocimene and the ‘mushroom-like’ *β*-ocimene were not present in the Apollo and Unique essential oil, respectively. Thus, these compounds could be used to differentiate essential oils from different feijoa cultivars.

Furthermore, the perceived aroma intensity of each compound also differed among the essential oils from the four cultivars. Some aroma active compounds were found to be specifically important in one or more cultivars. The ‘feijoa-like’ methyl benzoate and the ‘metallic’ *β*-myrcene were dominant aromatic compounds; both scored the highest aroma intensity in Apollo essential oils. The two compounds were also among the principle aromatic compounds in the Wiki Tu essential oil (aroma intensity at 3.67 and 4.33, respectively). Methyl benzoate was previously reported to be responsible for the characteristic ‘feijoa-like’ aroma [16], which is consistent with our olfactory result. The ‘herbal’ *δ*-cadinene contributed greatly to the aroma of the Opal Star essential oil (aroma intensity of 4.33) and also played an important role in the aroma of the Unique and Wiki Tu essential oil (aroma intensity at 3.33 and 3.67 respectively), while it was only detected to be ‘weak’ in the Apollo essential oil.

**Figure 5:**
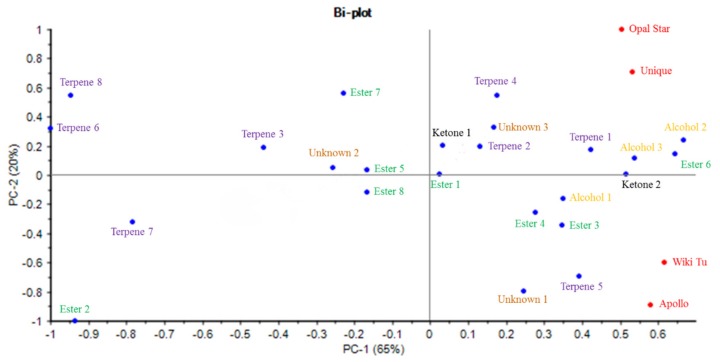
PCA analysis of aroma active compounds in feijoa essential oils from four feijoa cultivars. Compound labels are in accordance to their listing orders in each chemical group in Table 2.

The aroma active compounds were further subjected to a principle component analysis (PCA) to better evaluate the correlations among the four essential oils, as well as the associations between compounds and cultivars. As shown in Figure 5, the PCA model was successfully established, with a total of 85% variance extracted by the two principle components. The PC-1 and PC-2 expressed 65% and 20% of the variation, respectively.

All feijoa cultivars, located on the right side of the plot, show high correlations with those compounds separated to the same side by PC-1. This means 15 of the 24 identified aroma active compounds can be regarded as major contributors to the aroma of feijoa essential oils. Moreover, the PC-2 scale has further separated the four feijoa cultivars into different groups, with Opal Star and Unique in the upper side while Wiki Tu and Apollo in the lower side. This indicates that the Opal Star and Unique group can be better characterized by the 14 compounds in the upper side and the Wiki Tu and Apollo group had closer correlations to the eight compounds in the lower side.

More specifically, *δ*-cadinene, 2-undecanone, germacrene D, *α*-cubebene, *α*-terpineol, (*E*)-geraniol, (*Z*)-3-hexenyl butanoate, and the unknown compound (RI of 1367) contributed greatly to the aroma of the essential oils from Opal Star and Unique cultivars. On the other hand, the Apollo and Wiki Tu essential oils were strongly associated with linalool, methyl benzoate, ethyl benzoate, *β*-myrcene, and the unknown compound (RI of 1153). It is also noted that ethyl hexanoate (ester 1) and 3-octanone (ketone 2) were indifferent compounds in the aroma of the tested feijoa essential oils, as they are positioned on the boundary of PC-2.

It is well known that different cultivars of a fruit could have a significant impact on the aroma and flavor of the fruit [43,44]. Essential oils, containing concentrated aroma compounds, may further magnify the cultivar differences. Therefore, in the potential application of feijoa essential oils in the flavor and fragrance industries, cultivar selection may be of importance to maintain a standardized aroma experience.

### 2.5. Aroma Profile of Feijoa Essential Oil

Based on the identified aroma active compounds, five aroma attributes were further selected to establish the aroma profile of feijoa essential oil. They are ‘fruity and honey-like’, ‘herbal and woody’, ‘sweet and floral’, ‘metallic and pungent’, and ‘grassy and minty’ notes.

**Figure 6:**
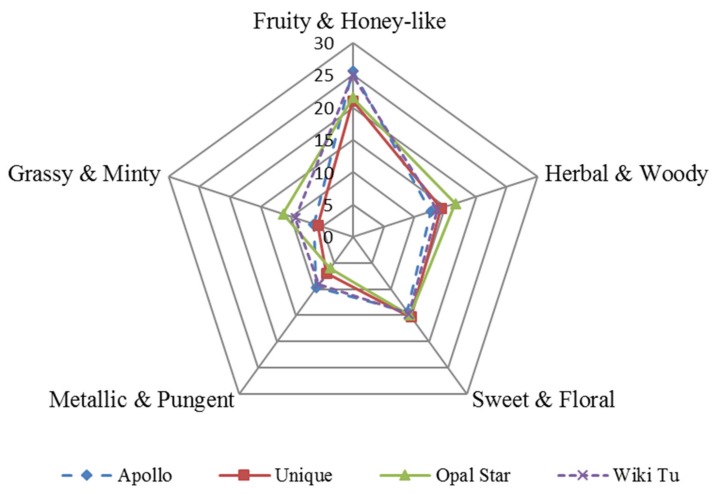
Aroma profile of feijoa essential oil from four cultivars.

As shown in Figure 6, essential oils extracted from the four different feijoa cultivars varied in the strength of each aroma attribute. The Apollo and Wiki Tu essential oils had stronger ‘fruity and honey-like’ and ‘metallic and pungent’ notes than the Unique and Opal Star essential oils, and the Opal Star essential oil was more ‘herbal and woody’ than the others. Regarding the ‘grassy and minty’ note, the Opal Star essential oil had the highest score, followed by the Wiki Tu essential oil and then the Apollo and Unique essential oils. However, no difference was observed in the strength of the ‘sweet and floral’ note among the four essential oils. Furthermore, the Opal Star essential oil was found to be more balanced in the aroma distribution of ‘fruity and honey-like’, ‘herbal and woody’, ‘sweet and floral’, and ‘grassy and minty’ notes, and was detected with less ‘metallic and pungent’ note, than the other essential oils. The Wiki Tu essential oil, which had the highest production yield (Figure 2), was dominated by the ‘fruity and honey-like’ note. In addition, the essential oil from the Unique cultivar was the least aroma intensive, as the enclosed area by the five attributes (Figure 6) was significantly smaller than the other three cultivars.

The ‘fruity and honey-like’ note was the leading attribute, with a higher score than the other attributes (Figure 6) in the aroma profile of feijoa essential oils. The typical ‘fruity and honey-like’ note was mostly formed by ester compounds including ethyl hexanoate, (*Z*)-3-hexenyl acetate, methyl benzoate, ethyl benzoate, and (*Z*)-3-hexenyl hexanoate, and the alcohol compound (*E*)-geraniol. To the contrary, the unpleasant ‘metallic and pungent’ note, bestowed by *β*-myrcene and the unknown compound (RI of 1153), was the weakest attribute in the aroma profile of feijoa essential oil. Therefore, the aroma property of feijoa essential oil predominantly possessed pleasant aromas and could be friendly to the flavor and fragrance industry to develop feijoa aroma-based products.

## 3. Materials and Methods

### 3.1. Feijoa Samples

Feijoa fruits of the Apollo, Unique, Opal Star, and Wiki Tu cultivars were supplied by local orchards in the northern island of New Zealand. Fresh fruits were collected during the commercial ripening season (March to June in New Zealand) and the peels of the fruits from each cultivar were obtained from the fresh fruit using a handle peeler. The peel samples were stored at −20 °C prior to experiments.

### 3.2. Chemicals

The following reference standards were purchased from Sigma-Aldrich (St. Louis, MO, USA): C7-C30 saturated alkane mixed standard, (internal standard) 2-methyl-3-heptanone, 2-methylpropyl 2-methylpropanoate, *β*-myrcene, *β*-trans-ocimene, ethyl hexanoate, 3-octanone, hexyl acetate, 2-methylbutyl 2-methylbutanoate, (*Z*)-3-hexenyl acetate, (*Z*)-hex-3-en-1-ol, 2-nonanone, 3-octanol, ethyl octanoate, (*Z*)-3-hexenyl butanoate, *α*-gurgujene, linalool, *β*-caryophyllene, (*Z*)-3-hexenyl (*E*)-2-butenoate, 2-undecanone, methyl benzoate, (*Z*)-3-hexenyl hexanoate, ethyl benzoate, humulene, *α*-farnesene, (*Z*)-3-hexenyl benzoate, and *β*-ocimene. All chemicals were of analytical grade or higher.

### 3.3. Extraction of Essential Oils from Feijoa Peel

Two traditional extraction methods, steam-distillation (SD) and hydro-distillation (HD) using a Clevenger-type apparatus [23], were employed to obtain essential oils from feijoa peels. Optimization of extraction time (for both SD and HD) and material to water ratio (for HD only) was carried out to maximize the extraction yield. Briefly, for SD, the whole apparatus was set on a hot plate with 800 g of fresh feijoa peel placed in the upper flask and 2 L of water in the nether flask. The water vapor produced by the boiling water carried the essential oils to the condenser and, subsequently, to the collector. Meanwhile, for HD, the peel and water were mixed in one flask with heating from the bottom. When the mixed solution was heated to boil point, the essential oils were produced along with the water vapor. After the vapor went through a condenser, the water phase and the oil phase automatically separated in the collector. The oil yield was measured and expressed as mL/kg fresh peel.

### 3.4. Compositional Analysis of Feijoa Essential Oil

The isolated essential oils were further dissolved in hexane and subjected to compositional analysis by gas chromatograph-mass spectrometry (GC-MS) (Shimadzu GCMS-QP2010 Plus, Kyoto, Japan). Briefly, 10 μL of pure essential oil was dissolved in 1 mL of hexane and a further 10 μL of 2-methyl-3-heptanone (312 ug/mL) was added as the internal standard. A DB-5MS column (30 m × 0.25 mm i.d., 0.25 μm film thickness (Agilent technologies, Inc., Santa Clara, CA, USA)) was employed. Helium (>99.99%) was used as the carrier gas at a flow rate of 1.5 mL/min. The injector temperature was 280 °C using a splitless injection mode with a sampling time of 1.00 min. The oven program was set as follows: Temperatures and times of 35 °C (0 min), 3 °C/min to 150 °C (5 min), 5 °C/min to 230 °C (5 min), and 20 °C/min to 280 °C (5 min). The electron-impact mass spectra were generated at 70 eV, with a scan range from 41 to 500 m/z, the ion source temperature was 200 °C, and the MS interface temperature was 250 °C.

The identification of the volatile compounds in feijoa essential oil was firstly conducted by the comparison of MS with the NIST Mass Spectral Library, through the Unknown Analysis software (Agilent Technologies, Inc, Santa Clara, CA, USA). These compounds were further confirmed with available reference analytical standards and literature records, as well as by comparing the linear retention index (LRI), calculated according to Van den Dool and Kratz [45], with that which was recorded in the NIST webbook (http://webbook.nist.gov/chemistry/) [46]. A semi-quantification of essential oil constituents was conducted according to Xiao et al. [30], relative to the internal standard 2-methyl-3-heptanone.

### 3.5. Aromatic Profile of Feijoa Essential Oil

The aromatic volatiles of feijoa essential oil were extracted, detected, and analyzed using headspace-solid phase micro extraction-GC-olfactory-MS (HS-SPME-GC-O-MS). In addition to the GC-MS system, an auto HS-SPME sampler controlled by a CombiPAL auto-sampler software (Cycle Composer Version 1.5.4, CTC Analytics AG, Zwingen Switzerland) and an olfactory port (Phaser OP275, ODP; ATAS/GL Sciences, Tokyo, Japan) connected via a 4-port splitter (SilFlow, Trajan, Australia) were equipped.

Briefly, 10 μL of pure essential oil was mixed with 1 mL of glycerol and placed in 20 mL amber glass screw-capped SPME vials (22.3 × 46mm, SUPELCO, PA, USA). Each vial was incubated in a shaking heated cube at 400 rpm for 25 min under 40 °C. The volatile compounds in feijoa essential oil were further extracted by a Divinylbenzene/Carboxen/Polydimethylsiloxane (DVB/CAR/PDMS) SPME fiber (50/30 μm film thickness and 1 cm length; Supelco, Bellefonte, PA, USA) for 5 min, and subjected to GC-MS analysis. The GC-MS program and conditions were as described above in Section 3.4. The GC column effluent was split to the MS and Olfactory port in a 1:2 ratio by a 4-port splitter (SilFlow, Trajan, Australia). The splitter was flushed with helium at 3 mL/min. The olfactory port transfer line was set at 220 °C. To prevent dryness of the nasal mucosa, humidified air was provided to the sniffing port at a rate of 20 mL/min.

For the detection of aromatic volatiles from feijoa essential oil, four trained panelists, consisting of two females and two males (aged 20–35), carried out the olfactory tests by sniffing through the olfactory port whilst the GC-MS was running. The retention time and the description and intensity of each detected aroma were recorded by the panelists during each test. An initial training using mixed natural aromatic compounds was conducted and the aroma description and intensity was standardized in trial experiments. Five scores, 1 (very weak), 2 (weak), 3 (medium), 4 (strong), and 5 (very strong), were used to evaluate the aroma intensity of each detected aromatic compound. The confirmation of aromatic compounds was made upon consistent detection by more than two panelists. The aroma profile was established after mutual agreement of all panelists. All samples were tested in triplicate.

### 3.6. Data Analysis

All experiments were conducted in triplicate. Statistical significance (*p* < 0.05) was determined by one-way ANOVA analysis using SPSS 22.0 (IBM Corp., Armonk, NY, USA). Principal component analysis (PCA) was conducted using the Unscrambler software (Version X 10.4, CAMO ASA, Norway).

## 4. Conclusions

The extraction of feijoa essential oil was optimized using SD and HD and the volatile and aroma active compounds the essential oils from four New Zealand grown cultivars were characterized by GC-MS and HS-SPME-GC-O-MS, and aroma profiles of feijoa essential oils, with a comparison of the four cultivars, were established. HD, with a material to water ratio of 1:4 and an extraction time of 90 min, was the optimized extraction method for feijoa essential oil. The Wiki Tu cultivar produced the highest essential oil yield among the four selected cultivars, which could potentially be applied in commercial feijoa essential oil production. A total of 160 compounds were detected, among which 90 compounds, novel to feijoa essential oils, were reported. Significant differences were observed in the concentrations of the detected compounds, as well as the aroma intensity of the aroma active compounds, both of which could potentially serve as bio-markers to differentiate feijoa cultivars. PCA results revealed that the essential oils from the Opal Star and Unique cultivars varied from the Wiki Tu and Apollo essential oils, with regards to their correlations to aroma active compounds. Terpenes and esters were dominant compounds in feijoa essential oil composition and were also major contributors to feijoa essential oil aroma. Five aroma attributes, ‘fruity and honey-like’, ‘herbal and woody’, ‘sweet and floral’, ‘metallic and pungent’, and ‘grassy and minty’, were selected to generate the aroma profile of feijoa essential oils, among which, the ‘fruity and honey-like’ note had the largest impact on the overall aroma of feijoa essential oils. Among the four essential oils, differences were observed in the strength of each aroma attribute. This study is the first to provide evidence on the yield of essential oils, compositional difference, and aroma profile diversity of feijoa essential oils from different cultivars. However, further exploration would be required to reveal the unknown compounds detected in the essential oils and additional feijoa cultivars could be tested for commercial production of essential oils.

## Figures and Tables

**Table 1:** Chemical Composition of feijoa essential oil from four cultivars.

No.	Name	CAS	RI ^a^ (RI ^b^)	MF	*C*	ID	Relative Concentration (10^−1^ mg/mL)			
							Apollo	Unique	Opal Star	Wiki Tu
**1**	Cyclohexylmethane	108-87-2	707 (720)	96.8	H	MS, RI	0.44 ± 0.03 ^a^	0.47 ± 0.07 ^a^	0.42 ± 0.01 ^a^	0.53 ± 0.01 ^b^
**2**	(*Z*)-Hex-3-en-1-ol	928-96-1	838 (855)	96.1	C	Std, MS, RI, LR	0.17 ± 0.004 ^b^	nd	0.39 ± 0.01 ^c^	0.01 ± 0.00 ^a^
**3**	2-Methylpropyl 2-methylpropanoate *	97-85-8	904 (906)	98.7	A	Std, MS, RI	3.15 ± 0.05 ^c^	0.20 ± 0.01 ^b^	0.22 ± 0.01 ^b^	0.07 ± 0.01 ^a^
**4**	Linalool 3,7-oxide *	7392-19-0	968 (969)	95.5	I	MS, RI	4.90 ± 0.17 ^a^	9.66 ± 0.04 ^b^	10.62 ± 0.58 ^b^	4.87 ± 0.11 ^a^
**5**	1-Octene-3-one *	4312-99-6	973 (975)	89.1	E	MS, RI	0.59 ± 0.02 ^ab^	0.64 ± 0.07 ^b^	1.73 ± 0.07 ^c^	0.34 ± 0.02 ^a^
**6**	3-Octanone *	106-68-3	983 (988)	98.9	E	Std, MS, RI, LR	87.61 ± 1.46 ^b^	7.77 ± 0.33 ^a^	236.41 ± 9.68 ^c^	12.89 ± 0.18 ^a^
**7**	*β*-Myrcene *	123-35-3	988 (993)	97.4	F	Std, MS, RI, LR	14.03 ± 0.13 ^b^	4.38 ± 0.05 ^a^	23.17 ± 1.27 ^c^	2.83 ± 0.10 ^a^
**8**	3-Octanol *	589-98-0	995 (996)	94.1	C	Std, MS, RI, LR	4.26 ± 0.10 ^a^	2.38 ± 0.002 ^a^	33.98 ± 1.74 ^b^	3.12 ± 0.13 ^a^
**9**	Ethyl hexanoate *	123-66-0	997 (998)	96.1	A	Std, MS, RI, LR	0.09 ± 0.01 ^a^	nd	18.87 ± 0.90 ^b^	nd
**10**	*δ*-Carene *	29050-33-7	999 (1001)	92.6	F	MS, RI	0.08 ± 0.01 ^a^	0.28 ± 0.005 ^c^	0.15 ± 0.01 ^b^	nd
**11**	2-Methylpropyl 2-methylbutanoate *	2445-67-2	1001 (1004)	97.9	A	MS, RI	18.40 ± 0.12 ^d^	4.18 ± 0.06 ^c^	2.54 ± 0.09 ^b^	0.40 ± 0.001 ^a^
**12**	(*Z*)-3-Hexenol acetate *	3681-71-8	1003 (1011)	99.1	A	Std, MS, RI, LR	7.90 ± 0.05 ^b^	nd	35.10 ± 1.70 ^c^	0.88 ± 0.01 ^a^
**13**	Hexyl acetate *	142-92-7	1010 (1012)	97.6	A	Std, MS, RI	1.28 ± 0.02 ^a^	nd	5.47 ± 0.25 ^b^	nd
**14**	3-Methylbutyl 2-methylpropanoate *	2050-01-3	1015 (1015)	98.7	A	MS, RI	32.24 ± 0.34 ^c^	7.04 ± 0.06 ^b^	7.16 ± 0.18 ^b^	1.22 ± 0.003 ^a^
**15**	*α*-Terpinene *	99-86-5	1018 (1020)	96.2	F	MS, RI	0.94 ± 0.02 ^b^	0.92 ± 0.04 ^b^	1.87 ± 0.05 ^c^	0.60 ± 0.04 ^a^
**16**	*o*-Cymene *	527-84-4	1025 (1018)	93.8	D	MS, RI	0.63 ± 0.08 ^b^	0.90 ± 0.02 ^c^	1.45 ± 0.12 ^d^	0.28 ± 0.02 ^a^
**17**	Limonene *	138-86-3	1030 (1028)	98.2	F	MS, RI, LR	12.21 ± 0.68 ^b^	9.39 ± 0.02 ^b^	20.48 ± 0.98 ^c^	5.40 ± 0.15 ^a^
**18**	(*E*)-*β*-Ocimene *	3779-61-1	1037 (1041)	99.2	F	Std, MS, RI, LR	121.67 ± 2.63 ^b^	105.72 ± 0.24 ^b^	201.49 ± 11.89 ^c^	27.89 ± 0.30 ^a^
**19**	*β*-Ocimene *	13877-91-3	1048 (1054)	98.8	F	Std, MS, RI	193.61 ± 4.65 ^c^	60.29 ± 0.43 ^b^	208.96 ± 11.07 ^c^	26.90 ± 0.21 ^a^
**20**	*γ*-Terpinene *	99-85-4	1060 (1062)	97.1	F	MS, RI	1.05 ± 0.01 ^a^	1.03 ± 0.01 ^a^	1.84 ± 0.11 ^b^	0.77 ± 0.02 ^a^
**21**	Unknown *	-	1061 (NA)	-	-	-	41.56 ± 0.47 ^d^	23.82 ± 1.43 ^c^	15.77 ± 1.08 ^b^	4.01 ± 0.02 ^a^
**22**	Acetophenone	98-86-2	1065 (1068)	95.3	E	MS, RI, LR	0.12 ± 0.002 ^a^	0.07 ± 0.01 ^a^	0.31 ± 0.02 ^b^	nd
**23**	3-Nonanone	925-78-0	1085 (1082)	92.9	E	MS, RI	0.14 ± 0.003 ^b^	nd	0.53 ± 0.02 ^c^	0.04 ± 0.00 ^a^
**24**	*α*-Terpinolen *	586-62-9	1087 (1085)	97.9	F	MS, RI	21.25 ± 0.33 ^b^	19.88 ± 0.11 ^b^	36.76 ± 2.02 ^c^	13.43 ± 0.08 ^a^
**25**	2-Nonanone *	821-55-6	1090 (1091)	97.9	E	Std, MS, RI, LR	8.91 ± 0.09 ^b^	3.89 ± 0.02 ^a^	13.89 ± 0.74 ^c^	2.60 ± 0.03 ^a^
**26**	*p*-Cymenene	1195-32-0	1090 (1090)	90.9	D	MS, RI	nd	0.83 ± 0.03 ^c^	0.52 ± 0.05 ^b^	0.25 ± 0.003 ^a^
**27**	Methyl benzoate *	93-58-3	1094 (1094)	97.8	A	Std, MS, RI, LR	82.92 ± 1.88 ^b^	2.40 ± 0.05 ^a^	154.62 ± 8.07 ^c^	17.03 ± 0.15 ^a^
**28**	Linalool *	78-70-6	1102 (1108)	99.2	C	Std, MS, RI, LR	287.1 ± 7.98 ^b^	28.65 ± 0.14 ^a^	532.42 ± 27.73 ^c^	25.81 ± 0.23 ^a^
**29**	2-Methylbutyl 2-methylbutanoate *	2445-78-5	1103 (1106)	91.7	A	Std, MS, RI, LR	36.99 ± 1.96 ^c^	17.06 ± 2.04 ^b^	28.49 ± 3.61 ^c^	2.31 ± 0.09 ^a^
**30**	Nonanal *	124-19-6	1104 (1102)	90.7	G	MS, RI, LR	3.78 ± 0.29 ^b^	14.9 ± 0.52 ^d^	9.15 ± 0.23 ^c^	0.46 ± 0.00 ^a^
**31**	Butyl 2-ethylbutyrate *	5129-48-6	1105 (1093)	86.6	A	MS, RI	nd	2.04 ± 0.05 ^b^	nd	0.48 ± 0.06 ^a^
**32**	Myrcenol	543-39-5	1119 (1123)	93.3	C	MS, RI	0.41 ± 0.04 ^a^	0.74 ± 0.04 ^b^	0.75 ± 0.00 ^b^	1.63 ± 0.07 ^c^
**33**	(*E,E*)-Cosmene *	460-01-5	1121 (1130)	90.8	H	MS, RI	0.46 ± 0.02 ^b^	0.15 ± 0.01 ^a^	0.62 ± 0.08 ^b^	0.01 ± 0.001 ^a^
**34**	(4*E*,6*Z*)-allo-Ocimene *	7216-56-0	1129 (1133)	98.7	F	MS, RI	27.58 ± 0.94 ^b^	22.76 ± 0.2 ^b^	43.72 ± 2.15 ^c^	6.58 ± 0.04 ^a^
**35**	(3*Z*)-3-Hexenyl 2-methylpropanoate *	41519-23-7	1141 (1145)	96.8	A	MS, RI	1.60 ± 0.08 ^b^	0.97 ± 0.09 ^a^	1.48 ± 0.10 ^b^	0.79 ± 0.004 ^a^
**36**	Unknown *	-	1153 (NA)	-	-	-	19.68 ± 0.67 ^c^	26.15 ± 0.18 ^d^	11.89 ± 0.93 ^b^	6.11 ± 0.57 ^a^
**37**	(2*E*)-2-Nonenal *	18829-56-6	1159 (1156)	96.3	G	MS, RI	0.61 ± 0.03 ^b^	0.53 ± 0.02 ^b^	1.10 ± 0.07 ^c^	0.12 ± 0.00 ^a^
**38**	Ocimenol *	5986-38-9	1164 (1174)	94.9	C	MS, RI	2.96 ± 0.34 ^a^	2.93 ± 0.17 ^a^	2.77 ± 0.12 ^a^	6.78 ± 0.06 ^b^
**39**	Ethyl benzoate *	93-89-0	1171 (1178)	98.3	A	Std, MS, RI, LR	31.21 ± 0.72 ^b^	1.25 ± 0.02 ^a^	182.97 ± 7.72 ^c^	1.01 ± 0.02 ^a^
**40**	(*Z*)-3-Hexenyl butanoate *	16491-36-4	1185 (1184)	97.5	A	Std, MS, RI, LR	69.63 ± 1.40 ^b^	17.06 ± 0.02 ^a^	213.82 ± 9.53 ^c^	27.28 ± 0.13 ^a^
**41**	3-Decanone *	928-80-3	1186 (1187)	93.0	E	MS, RI	7.85 ± 0.12 ^b^	4.91 ± 0.02 ^a^	19.35 ± 1.18 ^c^	4.27 ± 0.02 ^a^
**42**	Hexyl butanoate *	2639-63-6	1190 (1190)	97.7	A	MS, RI	3.95 ± 0.07 ^b^	1.87 ± 0.02 ^a^	11.88 ± 0.63 ^c^	1.20 ± 0.01 ^a^
**43**	2-Decanone *	693-54-9	1190 (1190)	97.8	E	MS, RI	4.84 ± 0.08 ^d^	2.72 ± 0.04 ^b^	4.15 ± 0.09 ^c^	1.05 ± 0.14 ^a^
**44**	Ethyl octanoate *	106-32-1	1194 (1191)	96.7	A	Std, MS, RI	0.07 ± 0.01 ^a^	nd	3.99 ± 0.18 ^b^	nd
**45**	*α*-Terpineol *	98-55-5	1197 (1199)	98.2	C	MS, RI, LR	39.52 ± 0.71 ^b^	12.96 ± 0.05 ^a^	69.87 ± 3.39 ^c^	38.08 ± 0.38 ^b^
**46**	*γ*-Terpineol	586-81-2	1201 (1201)	97.7	C	MS, RI	0.12 ± 0.01 ^a^	0.83 ± 0.02 ^b^	0.15 ± 0.02 ^a^	2.65 ± 0.06 ^c^
**47**	2-Heptyl butanoate *	39026-94-3	1209 (1199)	95.4	A	MS, RI, LR	4.14 ± 0.01 ^c^	1.51 ± 0.01 ^b^	1.11 ± 0.09 ^a^	1.44 ± 0.08 ^b^
**48**	*α*-Ionene	475-03-6	1215 (1255)	95.3	D	MS, RI	0.92 ± 0.04 ^c^	0.49 ± 0.02 ^b^	1.93 ± 0.08 ^d^	0.23 ± 0.004 ^a^
**49**	Carvomenthenal *	29548-14-9	1220 (1217)	94.5	G	MS, RI	0.85 ± 0.02 ^a^	0.74 ± 0.12 ^a^	4.07 ± 0.28 ^b^	1.35 ± 0.29 ^a^
**50**	(*Z*)-Geraniol	106-25-2	1224 (1220)	99.2	C	MS, RI, LR	9.06 ± 0.21 ^b^	1.70 ± 0.09 ^a^	15.74 ± 0.96 ^c^	3.30 ± 0.001 ^a^
**51**	(*Z*)-3-Hexenyl 3-methylbutanoate *	35154-45-1	1230 (1235)	94.2	A	MS, RI	1.50 ± 0.07 ^b^	0.43 ± 0.01 ^a^	2.40 ± 0.11 ^c^	0.33 ± 0.03 ^a^
**52**	(*Z*)-3-Hexenyl (*E*)-2-butenoate *	65405-80-3	1234 (NA)	98.4	A	Std, MS	2.69 ± 0.03 ^b^	0.22 ± 0.01 ^a^	5.72 ± 0.24 ^c^	0.62 ± 0.01 ^a^
**53**	*β*-Citral	106-26-3	1238 (1241)	91.1	G	MS, RI	0.36 ± 0.03 ^a^	nd	0.5 ± 0.01 ^b^	nd
**54**	octan-4-yl butanoate *	20286-46-8	1244 (NA)	81.7	A	MS	18.80 ± 0.24 ^b^	22.15 ± 0.09 ^c^	16.67 ± 1.28 ^b^	8.32 ± 0.08 ^a^
**55**	(*E*)-geraniol *	106-24-1	1250 (1253)	99.4	C	MS, RI, LR	34.64 ± 0.73 ^c^	5.55 ± 0.02 ^a^	57.43 ± 3.20 ^d^	12.47 ± 0.11 ^b^
**56**	(*S*)-(-)-Citronellic acid, methyl ester *	1000333-55-1	1256 (NA)	95.1	A	MS	0.36 ± 0.01 ^a^	0.24 ± 0.02 ^a^	1.60 ± 0.15 ^b^	0.18 ± 0.01 ^a^
**57**	Propyl benzoate	2315-68-6	1271 (1284)	96.2	A	MS, RI	0.58 ± 0.03 ^c^	nd	0.43 ± 0.01 ^b^	0.07 ± 0.001 ^a^
**58**	Unknown *	-	1275 (NA)	-	-	-	13.15 ± 0.27 ^c^	10.05 ± 0.32 ^b^	31.22 ± 0.03 ^d^	6.77 ± 0.52 ^a^
**59**	(*Z*)-3-Hexenyl pentanoate *	35852-46-1	1281 (1270)	96.1	A	MS, RI	1.04 ± 0.05 ^a^	0.60 ± 0.09 ^a^	2.95 ± 0.26 ^b^	0.3 ± 0.01 ^a^
**60**	Tricyclo[3.2.1.02,7]oct-3-ene, 2,3,4,5-tetramethyl-	62338-44-7	1284 (NA)	89.9	H	MS	0.22 ± 0.05 ^a^	0.64 ± 0.02 ^b^	nd	0.27 ± 0.01 ^a^
**61**	2-Undecanone *	112-12-9	1294 (1296)	97.3	E	Std, MS, RI, LR	196.97 ± 5.18 ^b^	220.36 ± 1.90 ^b^	245.43 ± 15.21 ^c^	125.93 ± 0.87 ^a^
**62**	Methyl geranate *	2349-14-6	1321 (1328)	95.6	A	MS, RI, LR	72.48 ± 2.10 ^b^	30.8 ± 4.91 ^a^	127.59 ± 10.70 ^c^	38.98 ± 0.12 ^a^
**63**	Elixene *	3242-08-8	1328 (1492)	90.5	B	MS, RI	10.56 ± 0.17 ^b^	11.57 ± 0.01 ^b^	15.14 ± 0.70 ^c^	6.32 ± 0.17 ^a^
**64**	2-methylpropyl benzoate *	120-50-3	1330 (1331)	95.1	A	MS, RI	7.75 ± 0.28 ^b^	2.42 ± 0.03 ^a^	3.35 ± 0.57 ^a^	2.74 ± 0.18 ^a^
**65**	*γ*-Elemene *	339154-91-5	1339 (NA)	95.5	B	MS	162.78 ± 6.94 ^b^	176.51 ± 2.37 ^b^	207.09 ± 8.41 ^c^	98.9 ± 1.49 ^a^
**66**	*α*-Cubebene *	17699-14-8	1354 (1359)	97.4	B	MS, RI, LR	187.05 ± 6.41 ^a^	337.15 ± 2.65 ^c^	219.62 ± 11.83 ^b^	180.91 ± 0.10 ^a^
**67**	Dehydro-ar-ionene *	30364-38-6	1360 (1359)	88.8	D	MS, RI	0.96 ± 0.01 ^a^	1.09 ± 0.03 ^a^	1.06 ± 0.09 ^a^	1.09 ± 0.02 ^a^
**68**	Unknown *	-	1367 (NA)	-	-	-	2.9 ± 0.07 ^a^	9.16 ± 0.13 ^b^	2.66 ± 0.67 ^a^	3.99 ± 0.11 ^a^
**69**	Methyl *p*-methoxybenzoate	121-98-2	1374 (1373)	95.8	A	MS, RI, LR	3.29 ± 0.01 ^b^	0.32 ± 0.02 ^a^	8.50 ± 0.50 ^c^	1.29 ± 0.07 ^a^
**70**	Isoledene *	95910-36-4	1378 (NA)	95.1	B	MS	22.51 ± 0.42 ^a^	43.61 ± 1.61 ^c^	20.31 ± 0.72 ^a^	36.99 ± 0.55 ^b^
**71**	(*Z*)-3-Hexenyl hexanoate *	31501-11-8	1381 (1384)	97.6	A	Std, MS, RI, LR	140.84 ± 3.91 ^c^	22.83 ± 0.31 ^a^	359.77 ± 11.75 ^d^	54.72 ± 0.02 ^b^
**72**	*α*-Copaene *	3856-25-5	1383 (1387)	95.4	B	MS, RI, LR	54.95 ± 2.25 ^a^	116.33 ± 2.63 ^c^	63.69 ± 2.39 ^a^	84.51 ± 1.30 ^b^
**73**	Hexyl hexanoate *	6378-65-0	1386 (1387)	90.8	A	MS, RI	5.60 ± 0.26 ^a^	nd	15.65 ± 0.64 ^b^	nd
**74**	*β*-Bourbonene *	5208-59-3	1393 (1397)	88.5	B	MS, RI, LR	26.01 ± 1.86 ^a^	146.55 ± 3.44 ^c^	36.52 ± 1.48 ^b^	45.49 ± 0.54 ^b^
**75**	*β*-Elemene *	515-13-9	1397 (1402)	98.1	B	MS, RI, LR	471.88 ± 17.29 ^a^	897.18 ± 34.75 ^b^	617.17 ± 38.60 ^a^	529.45 ± 2.01 ^a^
**76**	*α*-Gurgujene *	489-40-7	1417 (1423)	98.2	B	Std, MS, RI, LR	114.66 ± 4.06 ^a^	214.33 ± 0.59 ^c^	114.93 ± 5.97 ^a^	147.67 ± 0.10 ^b^
**77**	*β*-Cadinen	5951-61-1	1422 (NA)	98.5	B	MS	17.46 ± 0.51 ^a^	42.71 ± 1.05 ^c^	18.1 ± 0.81 ^a^	36.97 ± 1.05 ^b^
**78**	*β*-Caryophyllene *	87-44-5	1434 (1437)	99.6	B	Std, MS, RI, LR	1188.17 ± 74.80 ^ab^	1767.36 ± 51.36 ^c^	956.41 ± 59.64 ^a^	1314.09 ± 21.83 ^b^
**79**	*β*-Cubebene *	13744-15-5	1439 (1390)	96.8	B	MS, RI, LR	41.62 ± 1.40 ^b^	59.89 ± 4.60 ^c^	49.83 ± 2.89 ^bc^	39.53 ± 1.20 ^a^
**80**	Selina-3,7(11)-diene *	6813-21-4	1445 (1545)	95.4	B	MS, RI	10.08 ± 0.22 ^a^	33.6 ± 0.06 ^c^	10.78 ± 0.23 ^a^	29.48 ± 0.05 ^b^
**81**	*L*-Alloaromadendrene *	25246-27-9	1449 (1455)	98.3	B	MS, RI, LR	49.29 ± 0.58 ^a^	106.86 ± 8.47 ^b^	53.42 ± 3.58 ^a^	125.12 ± 4.93 ^b^
**82**	(*Z*)-*β*-Farnesene *	28973-97-9	1456 (1460)	94.2	B	MS, RI	76.34 ± 5.36 ^b^	48.61 ± 1.05 ^a^	46.94 ± 4.92 ^a^	54.42 ± 0.56 ^a^
**83**	Humulene *	6753-98-6	1469 (NA)	97.0	B	Std, MS, LR	626.06 ± 34.33 ^a^	1127.69 ± 97.72 ^b^	411.17 ± 19.04 ^a^	590.18 ± 76.59 ^a^
**84**	*β*-Gurjunene *	17334-55-3	1472 (NA)	97.7	B	MS, LR	81.12 ± 2.36 ^a^	165.15 ± 5.88 ^c^	102.64 ± 5.95 ^a^	127.61 ± 2.40 ^b^
**85**	*β*-Cadinene	523-47-7	1483 (1493)	94.5	B	MS, RI, LR	275.88 ± 11.86 ^b^	541.38 ± 3.96 ^d^	175.78 ± 14.12 ^a^	471.92 ± 2.65 ^c^
**86**	Germacrene D *	23986-74-5	1496 (1489)	94.5	B	MS, RI, LR	589.77 ± 26.79 ^a^	1091.86 ± 50.57 ^b^	1284.56 ± 90.86 ^b^	887.79 ± 120.79 ^ab^
**87**	2-Tridecanone *	593-08-8	1498 (1492)	94.5	E	MS, RI, LR	53.08 ± 0.75 ^b^	95.78 ± 2.96 ^c^	11.34 ± 0.69 ^a^	59.31 ± 0.10 ^b^
**88**	*α*-Farnesene *	502-61-4	1509 (1507)	92.4	B	Std, MS, RI, LR	450.84 ± 26.94 ^b^	622.59 ± 91.95 ^b^	502.99 ± 22.90 ^b^	188.42 ± 5.78 ^a^
**89**	Ledene *	21747-46-6	1510 (1495)	95.8	B	MS, RI, LR	1040.92 ± 148.68 ^a^	1919.90 ± 73.67 ^b^	1067.56 ± 172.45 ^a^	1554.67 ± 15.94 ^ab^
**90**	*γ*-Cadinene *	39029-41-9	1523 (1528)	94.7	B	MS, RI, LR	41.00 ± 0.29 ^a^	67.46 ± 1.60 ^c^	48.63 ± 2.08 ^b^	75.77 ± 0.61 ^d^
**91**	*δ*-Cadinene *	483-76-1	1529 (1537)	94.4	B	MS, RI, LR	481.16 ± 19.25 ^a^	651.52 ± 50.75 ^a^	562.50 ± 36.72 ^a^	692.32 ± 57.42 ^a^
**92**	Epizonarene	41702-63-0	1534 (1537)	91.5	B	MS, RI	335.62 ± 24.85 ^b^	155.09 ± 41.17 ^a^	115.48 ± 7.74 ^a^	143.90 ± 4.14 ^a^
**93**	Cadine-1,4-diene *	16728-99-7	1542 (1546)	95.5	B	MS, RI	89.77 ± 5.33 ^a^	188.09 ± 6.37 ^c^	106.73 ± 11.74 ^ab^	130.35 ± 0.26 ^b^
**94**	*α*-Cadinene *	24406-05-1	1546 (1552)	96.9	B	MS, RI	18.04 ± 1.40 ^a^	40.58 ± 1.78 ^c^	23.01 ± 0.62 ^a^	31.34 ± 0.12 ^b^
**95**	*α*-Calacorene	1000293-02-3	1551 (NA)	89.2	B	MS, LR	69.89 ± 2.16 ^bc^	73.44 ± 1.71 ^c^	59.38 ± 2.99 ^b^	45.14 ± 1.58 ^a^
**96**	Nerolidol	142-50-7	1570 (1565)	96.6	C	MS, RI	nd	379.77 ± 5.85 ^c^	72.65 ± 1.52 ^a^	172.63 ± 2.90 ^b^
**97**	*E*-Nerolidol	40716-66-3	1576 (1564)	90.8	C	MS, RI	186.05 ± 18.28 ^b^	95.40 ± 1.35 ^a^	nd	nd
**98**	(*Z*)-3-Hexenyl benzoate *	25152-85-6	1578 (1573)	99.0	A	Std, MS, RI, LR	335.29 ± 5.49 ^d^	81.06 ± 2.39 ^a^	255.33 ± 0.15 ^c^	171.31 ± 0.14 ^b^
**99**	Palustrol	5986-49-2	1585 (1579)	95.9	C	MS, RI, LR	nd	189.21 ± 2.63 ^a^	361.93 ± 12.07 ^b^	220.72 ± 14.7 ^a^
**100**	Hexyl benzoate *	6789-88-4	1589 (1596)	95.4	A	MS, RI, LR	nd	nd	44.47 ± 5.20	nd
**101**	Espatulenol	6750-60-3	1599 (1577)	91.8	C	MS, RI, LR	837.00 ± 26.69 ^b^	532.01 ± 0.34 ^ab^	599.69 ± 110.25 ^ab^	444.8 ± 40.44 ^a^
**102**	Unknown	-	1608 (NA)	-	-	-	518.31 ± 9.87 ^a^	870.52 ± 2.99 ^b^	546.46 ± 59.32 ^a^	1259.62 ± 32.07 ^c^
**103**	Unknown	-	1616 (NA)	-	-	-	339.65 ± 14.23 ^a^	661.31 ± 128.16 ^a^	320.03 ± 16.30 ^a^	847.53 ± 174.78 ^a^
**104**	Unknown	-	1619 (NA)	-	-	-	222.35 ± 3.04 ^a^	378.6 ± 4.87 ^c^	185.2 ± 9.23 ^a^	277.17 ± 15.84 ^b^
**105**	Rosifoliol	63891-61-2	1625 (1613)	92.4	C	MS, RI, LR	218.95 ± 3.27 ^a^	521.22 ± 33.27 ^b^	251.60 ± 12.25 ^a^	673.08 ± 58.05 ^b^
**106**	Unknown	-	1634 (NA)	-	-	-	31.34 ± 0.25 ^a^	71.50 ± 1.49 ^c^	52.14 ± 1.50 ^b^	92.76 ± 3.89 ^d^
**107**	(1-Butylheptyl)benzene	4537-15-9	1636 (1631)	87.1	D	MS, RI	nd	nd	28.86 ± 1.86 ^b^	15.20 ± 0.63 ^a^
**108**	(−)-Spathulenol *	77171-55-2	1649 (1619)	93.9	C	MS, RI	531.01 ± 23.07 ^ab^	604.92 ± 5.17 ^b^	598.38 ± 43.44 ^b^	450.36 ± 6.82 ^a^
**109**	Unknown *	-	1658 (NA)	-	-	-	483.13 ± 19.00 ^b^	541.56 ± 5.46 ^c^	393.40 ± 0.49 ^a^	529.95 ± 0.90 ^bc^
**110**	*γ*-Eudesmol *	1209-71-8	1669 (1652)	84.2	C	MS, RI	40.24 ± 0.11 ^a^	nd	nd	65.14 ± 0.89 ^b^
**111**	*α*-Cadinol	481-34-5	1672 (1668)	96.1	C	MS, RI, LR	622.16 ± 23.31 ^a^	754.43 ± 23.41 ^a^	809.79 ± 41.07 ^ab^	1057.76 ± 96.91 ^b^
**112**	Unknown *	-	1677 (NA)	-	-	-	140.30 ± 26.44 ^b^	180.56 ± 6.16 ^b^	64.52 ± 6.53 ^a^	311.02 ± 9.75 ^c^
**113**	Cadalene *	483-78-3	1686 (1672)	86.2	D	MS, RI	nd	14.46 ± 1.36 ^a^	nd	11.23 ± 3.00 ^a^
**114**	Unknown	-	1703 (NA)	-	-	-	48.53 ± 2.62 ^a^	72.62 ± 0.70 ^b^	49.35 ± 2.49 ^a^	48.72 ± 2.15 ^a^
**115**	(1-Methyldecyl)benzene *	4536-88-3	1706 (1692)	93.4	D	MS, RI	nd	nd	25.55 ± 1.44 ^b^	10.19 ± 0.24 ^a^
**116**	Unknown	-	1723 (NA)	-	-	-	597.59 ± 17.41 ^c^	398.47 ± 48.79 ^b^	245.44 ± 17.97 ^a^	459.2 ± 9.26 ^b^
**117**	Unknown	-	1729 (NA)	-	-	-	54.73 ± 2.03 ^a^	117.16 ± 0.52 ^b^	47.53 ± 0.90 ^a^	105.23 ± 17.08 ^b^
**118**	(1-Pentylheptyl)benzene *	2719-62-2	1730 (1737)	90.9	D	MS, RI	nd	nd	84.85 ± 9.26 ^a^	65.59 ± 0.87 ^a^
**119**	(1-Butyloctyl)benzene *	2719-63-3	1734 (1742)	96.7	D	MS, RI	nd	nd	79.41 ± 4.73 ^b^	41.49 ± 0.04 ^a^
**120**	*E,E*-Farnesal	502-67-0	1740 (1737)	88.4	G	MS, RI	18.84 ± 1.51 ^c^	9.97 ± 0.40 ^b^	0.71 ± 0.05 ^a^	6.21 ± 0.54 ^b^
**121**	(1-Propylnonyl)benzene *	2719-64-4	1746 (1755)	88.7	D	MS, RI	34.13 ± 0.57 ^a^	nd	59.23 ± 0.37 ^c^	52.58 ± 1.74 ^b^
**122**	Unknown	-	1763 (NA)	-	-	-	54.49 ± 1.19 ^ab^	61.94 ± 2.94 ^b^	46.46 ± 3.51 ^a^	44.10 ± 1.04 ^a^
**123**	3-Phenyldodecane *	2400-00-2	1770 (1776)	97.5	D	MS, RI	nd	nd	48.23 ± 2.39 ^b^	23.30 ± 0.65 ^a^
**124**	Benzyl Benzoate *	120-51-4	1776 (1765)	97.4	A	MS, RI, LR	142.81 ± 3.39 ^c^	27.10 ± 1.31 ^a^	80.16 ± 3.97 ^b^	134.00 ± 1.26 ^c^
**125**	Unknown	-	1789 (NA)	-	-	-	217.52 ± 4.15 ^d^	78.76 ± 0.49 ^b^	46.59 ± 2.42 ^a^	105.58 ± 0.10 ^c^
**126**	Unknown	-	1804 (NA)	-	-	-	77.73 ± 2.68 ^b^	72.53 ± 1.68 ^b^	70.31 ± 3.78 ^b^	8.06 ± 0.18 ^a^
**127**	2-Phenyldodecane *	2719-61-1	1809 (1810)	92.6	D	MS, RI	nd	nd	61.99 ± 3.18 ^b^	30.04 ± 0.95 ^a^
**128**	(1-Pentyloctyl)benzene *	4534-49-0	1827 (1826)	92.0	D	MS, RI	nd	nd	143.47 ± 10.13 ^b^	73.75 ± 1.03 ^a^
**129**	(1-Butylnonyl)benzene *	4534-50-3	1834 (1831)	93.5	D	MS, RI	4.84 ± 0.34 ^a^	6.38 ± 0.26 ^a^	103.58 ± 5.51 ^c^	50.31 ± 0.16 ^b^
**130**	Benzene, (1-propyldecyl)- *	4534-51-4	1847 (1841)	92.4	D	MS, RI	nd	nd	66.47 ± 0.83 ^b^	35.58 ± 0.25 ^a^
**131**	*β*-Phenylethyl benzoate	94-47-3	1860 (1859)	95.0	A	MS, RI	6.86 ± 0.27 ^b^	2.32 ± 0.01 ^a^	24.97 ± 1.39 ^c^	8.27 ± 0.12 ^b^
**132**	Methyl 8,11,14,17-eicosatetraenoate	1000336-47-0	1871 (NA)	86.5	A	MS	4.00 ± 0.05 ^a^	5.06 ± 0.06 ^b^	nd	nd
**133**	Benzene, (1-ethylundecyl)-	4534-52-5	1872 (1859)	89.6	D	MS, RI	nd	nd	61.23 ± 3.12 ^b^	34.37 ± 0.64 ^a^
**134**	Benzyl salicylate	118-58-1	1876 (1859)	94.5	A	MS, RI	6.87 ± 0.18 ^c^	1.42 ± 0.05 ^a^	7.57 ± 0.44 ^c^	2.88 ± 0.14 ^b^
**135**	2-Heptadecanone	2922-51-2	1900 (1890)	93.5	E	MS, RI	1.41 ± 0.05 ^b^	1.56 ± 0.06 ^b^	nd	0.70 ± 0.02 ^a^
**136**	Benzene, (1-methyldodecyl)-	4534-53-6	1912 (1922)	94.6	D	MS, RI	nd	nd	64.24 ± 2.52 ^b^	31.99 ± 0.49 ^a^
**137**	Butyl phthalate	84-74-2	1953 (1940)	94.5	A	MS, RI	1.40 ± 0.04 ^b^	nd	1.47 ± 0.06 ^b^	0.36 ± 0.03 ^a^
**138**	Hexadecanoic acid	57-10-3	1955 (1960)	90.9	J	MS, RI	nd	0.69 ± 0.08 ^a^	nd	1.41 ± 0.13 ^b^
**139**	Geranyl benzoate	1000133-42-4	1964 (NA)	98.1	A	MS	15.76 ± 0.45 ^b^	4.49 ± 0.10 ^a^	19.33 ± 1.86 ^b^	24.27 ± 0.46 ^c^
**140**	(3*E*)-3-Hexenyl laurate	1000159-94-3	1975 (NA)	94.9	A	MS	7.10 ± 0.86 ^b^	0.74 ± 0.09 ^a^	nd	nd
**141**	Ethyl hexadecanoate	628-97-7	1990 (1991)	94.9	A	MS, RI	4.91 ± 0.10 ^a^	nd	18.27 ± 0.81 ^b^	nd
**142**	Unknown	-	2021 (NA)	-	-	-	33.69 ± 1.37 ^c^	2.88 ± 0.01 ^a^	67.62 ± 3.36 ^d^	18.11 ± 0.2 ^b^
**143**	Dibenzoylmethane	120-46-7	2079 (NA)	96.8	E	MS	5.45 ± 0.44 ^ab^	8.28 ± 0.30 ^c^	4.77 ± 0.14 ^a^	6.37 ± 0.15 ^b^
**144**	Phytol	150-86-7	2108 (2103)	96.5	C	MS, RI	nd	11.05 ± 0.08	nd	nd
**145**	Ethyl (9*Z*,11*E*)-9,11-octadecadienoate	1000336-69-8	2159 (NA)	95.7	A	MS	5.45 ± 0.08 ^a^	nd	24.49 ± 1.31 ^b^	nd
**146**	Ethyl linolenate	1191-41-9	2165 (2166)	93.4	A	MS, RI	0.98 ± 0.06 ^a^	nd	7.58 ± 0.09 ^b^	nd
**147**	Ethyl Oleate	111-62-6	2171 (2179)	88.1	A	MS, RI	nd	nd	0.80 ± 0.07	nd
**148**	Flavone	525-82-6	2225 (2150)	98.1	-	MS, RI, LR	160.17 ± 4.76 ^b^	153.53 ± 0.46 ^b^	105.00 ± 5.85 ^a^	161.06 ± 0.61 ^b^
**149**	Tricosane	638-67-5	2299 (2300)	95.4	H	MS, RI	1.18 ± 0.04 ^b^	0.68 ± 0.02 ^a^	1.41 ± 0.05 ^c^	0.71 ± 0.02 ^a^
**150**	Fumaric acid, decyl trans-hex-3-enyl ester	1000348-89-0	2376 (NA)	82.7	A	MS	3.34 ± 0.08 ^b^	nd	1.74 ± 0.17 ^a^	1.68 ± 0.01 ^a^
**151**	Lignoceric alcohol	506-51-4	2394 (NA)	95.3	C	MS	2.06 ± 0.09 ^b^	1.08 ± 0.22 ^a^	2.33 ± 0.11 ^b^	0.93 ± 0.25 ^a^
**152**	Tetracosane	646-31-1	2400 (2400)	94.4	H	MS, RI	1.11 ± 0.03 ^c^	0.76 ± 0.01 ^b^	0.60 ± 0.05 ^ab^	0.53 ± 0.05 ^a^
**153**	Piperonyl butoxide	51-03-6	2403 (2407)	98.0	I	MS, RI	nd	nd	10.84 ± 0.68 ^c^	4.00 ± 0.03 ^b^
**154**	1-Hexacosene	18835-33-1	2595 (2596)	95.2	H	MS, RI	nd	1.93 ± 0.01 ^a^	3.00 ± 0.11 ^b^	nd
**155**	Tetracosanal	57866-08-7	2634 (2632)	91.9	G	MS, RI	6.70 ± 0.09 ^b^	1.39 ± 0.09 ^a^	7.81 ± 0.64 ^b^	2.99 ± 0.12 ^a^
**156**	Heptacosane	593-49-7	2700 (2700)	96.1	H	MS, RI	4.52 ± 0.38 ^b^	2.22 ± 0.07 ^a^	2.74 ± 0.05 ^a^	2.01 ± 0.16 ^a^
**157**	Unknown	-	2837 (NA)	-	-	-	21.00 ± 0.28 ^b^	4.92 ± 0.27 ^a^	20.59 ± 3.12 ^b^	8.24 ± 0.56 ^a^
**158**	Unknown	-	3040 (NA)	-	-	-	17.42 ± 1.09 ^c^	4.77 ± 0.08 ^a^	11.19 ± 0.63 ^b^	6.10 ± 0.22 ^a^
**Additional Compounds Only Detected in SPME-GC-MS**										
**1**	3-Octanyl acetate	4864-61-3	1125 (1131)	92.0	A	MS, RI	-			
**2**	*β*-Selinene	17066-67-0	1493 (1496)	93.4	B	MS, RI, LR	-			

RI ^a^—retention index calculated; RI ^b^—retention index from the NIST webbook database, NA—not recorded in database; *—compounds detected in solid phase micro extraction; *C*—compound classification, A—ester, B—sesquiterpene, C—alcohol, D—aromatic hydrocarbon, E—ketone, F—monoterpene, G—aldehyde, H—hydrocarbon, I—ether, J—acid; ID, compound identification methods, MS—confirmed by mass spectrum, RI—confirmed by retention index in database, Std—confirmed by reference standard, LR—confirmed by published literature [27,28]; nd—not detected; ^a, b, c, d^—indicators for statistical significance among cultivars (*p* < 0.05), identical letters indicate no statistically significant difference.

**Table 2:** Odor active volatiles in feijoa essential oil from four cultivars.

No.	Name	CAS	Odor Description	Average Intensity			
				Apollo	Unique	Opal Star	Wiki Tu
**Ester**							
**1**	Ethyl hexanoate	123-66-0	apple-like	3.00	3.33	2.83	3.33
**2**	(*Z*)-3-Hexenyl acetate	3681-71-8	banana-like	2.33	nd	nd	2.67
**3**	Methyl benzoate	93-58-3	feijoa -like	5.00	2.33	3.67	3.67
**4**	Ethyl benzoate	93-89-0	fresh and fruity	4.33	3.33	3.00	3.67
**5**	(*Z*)-3-Hexenyl butanoate	16491-36-4	grassy	2.33	1.67	3.33	3.33
**6**	(*Z*)-3-Hexenyl hexanoate	31501-11-8	honey-like	3.67	4.00	4.33	4.67
**7**	Octan-4-yl butanoate	20286-46-8	herbal	2.33	3.00	3.67	2.00
**8**	Methyl geranate	2349-14-6	bitter	3.00	2.33	2.67	3.00
**Alcohol**							
**9**	Linalool	78-70-6	floral	4.33	4.00	3.00	3.67
**10**	*α*-Terpineol	98-55-5	fresh and minty	4.00	4.00	4.67	4.33
**11**	(*E*)-geraniol	106-24-1	fruity and peach-like	4.00	4.67	3.67	4.00
**Ketone**							
**12**	2-Undecanone	112-12-9	bitter and herbal	3.00	3.33	3.33	3.00
**13**	3-Octanone	106-68-3	mushroom-like	4.33	3.67	4.00	4.00
**Terpene**							
**14**	*α*-Cubebene	17699-14-8	sweet and herbal	4.00	3.33	4.33	3.67
**15**	Germacrene D	23986-74-5	sweet, herbal, floral	3.00	3.67	3.33	3.33
**16**	*β*-Selinene	17066-67-0	woody	2.33	2.33	2.67	2.00
**17**	*δ*-Cadinene	483-76-1	herbal	2.00	3.33	4.33	3.67
**18**	*β*-Myrcene	123-35-3	metallic	5.00	3.33	2.33	4.33
**19**	(*E*)-*β*-Ocimene	3779-61-1	sweet and herbal	nd	1.67	1.67	1.67
**20**	*β*-Ocimene	13877-91-3	mushroom-like	2.33	nd	2.00	2.00
**21**	(4*E*,6*Z*)-allo-Ocimene	7216-56-0	fresh and minty	nd	nd	3.33	1.67
**Others**							
**22**	unknown (RI of 1153)	-	bitter and pungent	4.67	3.67	3.67	4.67
**23**	unknown (RI of 1275)	-	sweet and herbal	3.00	2.67	2.67	2.33
**24**	unknown (RI of 1367)	-	fruity and honey-like	3.33	3.33	4.00	3.00

nd—not detected.

## References

[1] Burt S. (2004). Essential oils: Their antibacterial properties and potential applications in foods—A review. Int. J. Food Microbiol..

[2] Bakkali F., Averbeck S., Averbeck D., Idaomar M. (2008). Biological effects of essential oils—A review. Food Chem. Toxicol..

[3] Edris A.E. (2007). Pharmaceutical and therapeutic potentials of essential oils and their individual volatile constituents: A review. Phytother. Res..

[4] Buckle J. (2014). Clinical Aromatherapy-E-Book: Essential Oils in Practice.

[5] Chouliara E., Karatapanis A., Savvaidis I., Kontominas M. (2007). Combined effect of oregano essential oil and modified atmosphere packaging on shelf-life extension of fresh chicken breast meat, stored at 4 °C. Food Microbiol..

[6] Kykkidou S., Giatrakou V., Papavergou A., Kontominas M., Savvaidis I. (2009). Effect of thyme essential oil and packaging treatments on fresh Mediterranean swordfish fillets during storage at 4 °C. Food Chem..

[7] Abdollahi M., Rezaei M., Farzi G. (2012). Improvement of active chitosan film properties with rosemary essential oil for food packaging. Int. J. Food Sci. Technol..

[8] Wen P., Zhu D.-H., Wu H., Zong M.-H., Jing Y.-R., Han S.-Y. (2016). Encapsulation of cinnamon essential oil in electrospun nanofibrous film for active food packaging. Food Control.

[9] Cavanagh H., Wilkinson J. (2002). Biological activities of lavender essential oil. Phytother. Res..

[10] Hirokawa K., Nishimoto T., Taniguchi T. (2012). Effects of lavender aroma on sleep quality in healthy Japanese students. Percept. Mot. Ski..

[11] Jo C., Park B.J., Chung S.H., Kim C.B., Cha B.S., Byun M.W. (2004). Antibacterial and anti-fungal activity of citrus (Citrus unshiu) essential oil extracted from peel by-products. Food Sci. Biotechnol..

[12] Chalova V.I., Crandall P.G., Ricke S.C. (2010). Microbial inhibitory and radical scavenging activities of cold-pressed terpeneless Valencia orange (Citrus sinensis) oil in different dispersing agents. J. Sci. Food Agric..

[13] Amorim J.L., Simas D.L.R., Pinheiro M.M.G., Moreno D.S.A., Alviano C.S., da Silva A.J.R., Fernandes P.D. (2016). Anti-inflammatory properties and chemical characterization of the essential oils of four citrus species. PLoS ONE.

[14] Yang C., Chen H., Chen H., Zhong B., Luo X., Chun J. (2017). Antioxidant and anticancer activities of essential oil from Gannan navel orange peel. Molecules.

[15] Sharpe R., Sherman W., Miller E. Feijoa history and improvement. Proceedings of the 106th Annual Meeting of the Florida State Horticultural Society.

[16] Hardy P., Michael B. (1970). Volatile components of feijoa fruits. Phytochemistry.

[17] Mehinagic E., Royer G., Symoneaux R., Jourjon F., Prost C. (2006). Characterization of odor-active volatiles in apples: Influence of cultivars and maturity stage. J. Agric. Food Chem..

[18] Ghaste M., Narduzzi L., Carlin S., Vrhovsek U., Shulaev V., Mattivi F. (2015). Chemical composition of volatile aroma metabolites and their glycosylated precursors that can uniquely differentiate individual grape cultivars. Food Chem..

[19] Šamec D., Maretić M., Lugarić I., Mešić A., Salopek-Sondi B., Duralija B. (2016). Assessment of the differences in the physical, chemical and phytochemical properties of four strawberry cultivars using principal component analysis. Food Chem..

[20] Renaud E.N., Charles D.J., Simon J.E. (2001). Essential oil quantity and composition from 10 cultivars of organically grown lavender and lavandin. J. Essent. Oil Res..

[21] Lan-Phi N.T., Shimamura T., Ukeda H., Sawamura M. (2009). Chemical and aroma profiles of yuzu (Citrus junos) peel oils of different cultivars. Food Chem..

[22] Rawat A., Tripathi R., Khan A., Balasubrahmanyam V. (1989). Essential oil components as markers for identification of *Piper betle* L. cultivars. Biochem. Syst. Ecol..

[23] Boutekedjiret C., Bentahar F., Belabbes R., Bessiere J. (2003). Extraction of rosemary essential oil by steam distillation and hydrodistillation. Flavour Fragr. J..

[24] Kasuan N., Yunus M., Rahiman M.H.F., Aris S.R.S., Taib M.N. Essential oil composition of Kaffir lime: Comparative analysis between controlled steam distillation and hydrodistillation extraction process. Proceedings of the 2009 IEEE Student Conference on Research and Development (SCOReD).

[25] Sefidkon F., Abbasi K., Khaniki G.B. (2006). Influence of drying and extraction methods on yield and chemical composition of the essential oil of Satureja hortensis. Food Chem..

[26] Babu K.G., Kaul V. (2005). Variation in essential oil composition of rose-scented geranium (*Pelargonium* sp.) distilled by different distillation techniques. Flavour Fragr. J..

[27] Shaw G.J., Allen J.M., Yates M.K. (1989). Volatile flavour constituents in the skin oil from *Feijoa sellowiana*. Phytochemistry.

[28] Fernandez X., Loiseau A.-M., Poulain S., Lizzani-Cuvelier L., Monnier Y. (2004). Chemical composition of the essential oil from feijoa (*Feijoa sellowiana* Berg.) peel. J. Essent. Oil Res..

[29] Xiao Z., Ma S., Niu Y., Chen F., Yu D. (2016). Characterization of odour-active compounds of sweet orange essential oils of different regions by gas chromatography-mass spectrometry, gas chromatography-olfactometry and their correlation with sensory attributes. Flavour Fragr. J..

[30] Xiao Z., Li Q., Niu Y., Zhou X., Liu J., Xu Y., Xu Z. (2017). Odor-active compounds of different lavender essential oils and their correlation with sensory attributes. Ind. Crop. Prod..

[31] Binder R.G., Flath R.A. (1989). Volatile components of pineapple guava. J. Agric. Food Chem..

[32] Shaw G.J., Allen J.M., Yates M.K., Franich R.A. (1990). Volatile flavour constituents of feijoa (*Feijoa sellowiana*)—analysis of fruit flesh. J. Sci. Food Agric..

[33] Shaw G.J., Ellingham P.J., Birch E.J. (1983). Volatile constituents of feijoa—headspace analysis of intact fruit. J. Sci. Food Agric..

[34] Fernandes E.S., Passos G.F., Medeiros R., da Cunha F.M., Ferreira J., Campos M.M., Pianowski L.F., Calixto J.B. (2007). Anti-inflammatory effects of compounds alpha-humulene and (−)-trans-caryophyllene isolated from the essential oil of Cordia verbenacea. Eur. J. Pharmacol..

[35] Dahham S., Tabana Y., Iqbal M., Ahamed M., Ezzat M., Majid A., Majid A. (2015). The anticancer, antioxidant and antimicrobial properties of the sesquiterpene *β*-caryophyllene from the essential oil of Aquilaria crassna. Molecules.

[36] Yao Y.-Q., Ding X., Jia Y.-C., Huang C.-X., Wang Y.-Z., Xu Y.-H. (2008). Anti-tumor effect of *β*-elemene in glioblastoma cells depends on p38 MAPK activation. Cancer Lett..

[37] Cheng S.-S., Wu C.-L., Chang H.-T., Kao Y.-T., Chang S.-T. (2004). Antitermitic and antifungal activities of essential oil of Calocedrus formosana leaf and its composition. J. Chem. Ecol..

[38] Huo M., Cui X., Xue J., Chi G., Gao R., Deng X., Guan S., Wei J., Soromou L.W., Feng H. (2013). Anti-inflammatory effects of linalool in RAW 264.7 macrophages and lipopolysaccharide-induced lung injury model. J. Surg. Res..

[39] Hamm S., Bleton J., Connan J., Tchapla A. (2005). A chemical investigation by headspace SPME and GC–MS of volatile and semi-volatile terpenes in various olibanum samples. Phytochemistry.

[40] Jirovetz L., Buchbauer G., Stoyanova A., Balinova A., Guangjiun Z., Xihan M. (2005). Solid phase microextraction/gas chromatographic and olfactory analysis of the scent and fixative properties of the essential oil of *Rosa damascena* L. from China. Flavour Fragr. J..

[41] Jetti R., Yang E., Kurnianta A., Finn C., Qian M. (2007). Quantification of selected aroma-active compounds in strawberries by headspace solid-phase microextraction gas chromatography and correlation with sensory descriptive analysis. J. Food Sci..

[42] Pripdeevech P., Khummueng W., Park S.-K. (2011). Identification of odor-active components of agarwood essential oils from Thailand by solid phase microextraction-GC/MS and GC-O. J. Essent. Oil Res..

[43] Forney C.F., Kalt W., Jordan M.A. (2000). The composition of strawberry aroma is influenced by cultivar, maturity, and storage. HortScience.

[44] Munafo Jr J.P., Didzbalis J., Schnell R.J., Schieberle P., Steinhaus M. (2014). Characterization of the major aroma-active compounds in mango (*Mangifera indica* L.) cultivars Haden, White Alfonso, Praya Sowoy, Royal Special, and Malindi by application of a comparative aroma extract dilution analysis. J. Agric. Food Chem..

[45] Van den Dool H., Kratz P.D. (1963). A generalization of the retention index system including linear temperature programmed gas—liquid partition chromatography. J. Chromatogr. A.

[46] Linstrom P.J., Mallard W.G. (2019). NIST Chemistry Webbook. NIST Standard Reference Database No. 69.

